# Advanced Basal Cell Carcinoma: A Narrative Review on Current Systemic Treatments and the Neoadjuvant Approach

**DOI:** 10.3390/jpm15060226

**Published:** 2025-06-01

**Authors:** Andrea Paradisi, Maria Mannino, Francesco Brunetti, Enrico Bocchino, Alessandro Di Stefani, Ketty Peris

**Affiliations:** 1 UOC di Dermatologia, Dipartimento di Scienze Mediche e Chirurgiche Addominali ed Endrocrino Metaboliche, Fondazione Policlinico Universitario A. Gemelli—IRCCS, 00168 Rome, Italyketty.peris@unicatt.it (K.P.); 2 Dermatologia, Dipartimento di Medicina e Chirurgia Traslazionale, Università Cattolica del Sacro Cuore, 00168 Rome, Italy

**Keywords:** advanced basal cell carcinoma, systemic therapy, hedgehog pathway inhibitors, immune-checkpoint inhibitors, neoadjuvant therapy

## Abstract

**Background/Objectives:** Systemic therapy with hedgehog pathway inhibitors (HHIs) and anti-programmed cell death protein 1 (PD-1) antibodies represent the first- and second-line treatment options for advanced basal cell carcinoma (aBCC), respectively. A shift in the treatment paradigms toward the neoadjuvant approach is gaining increasing interest in aBCC management, whereby prior systemic therapy followed by surgery is likely to yield more favorable outcomes. The aim of this narrative review is to summarize the current evidence on systemic treatment options and the neoadjuvant approach for aBCC management. **Methods:** We performed a non-systematic review of the literature based on PubMed as search engine. **Results:** The pivotal phase II trials ERIVANCE and BOLT investigated the efficacy and safety profile of vismodegib and sonidegib, respectively, with reported objective response rates (ORRs) of 60.3% and 56% in laBCC patients, respectively. The pivotal phase II trial NCT03132636 investigated the efficacy and safety profile of cemiplimab in patients who progressed or were intolerant to prior HHI therapy, with an ORR of 32.1% in laBCC patients. Real-life studies confirmed the effectiveness and safety profile of HHI and anti-PD-1 immunotherapy. Several phase I/II clinical trials are currently investigating HHIs and immune-checkpoint inhibitors in the neoadjuvant setting followed by surgery for aBCC patients, with the aim of providing more favorable treatment outcomes, especially when upfront surgery would result in functional and/or aesthetic sequelae. **Conclusions:** Advanced BCC treatment is challenging, and the neoadjuvant approach followed by surgery is expected to reduce surgical complexity, increase tissue preservation, and improve patients’ satisfaction.

## 1. Introduction

Basal cell carcinoma (BCC) is the most common cutaneous malignancy, and it accounts for 75% of non-melanoma skin cancers [1]. The true incidence of BCC is difficult to assess, as diagnoses are not routinely recorded in national cancer registries, and histopathological confirmation is not mandatory for BCCs managed by topical medical and/or destructive therapies [2]. In Europe, the incidence rate of BCC ranges from 76 to 165 new diagnoses per 100,000 inhabitants [3]. Ultra-violet (UV) light exposure, increasing age, male sex, fair pigmentary characteristics, and immunosuppression represent the most important risk factors for BCC development [2,4].

The majority of BCCs are diagnosed in a localized and early stage and can be successfully managed by surgery and topical medical and/or destructive therapies [2,5]. Advanced BCC (aBCC), which includes locally advanced (la) and metastatic (m) BCCs, accounts for less than 10% of all BCC diagnoses [2]. The term advanced BCC encompasses a complex and heterogeneous group of lesions for which curative surgery and/or radiation is not possible due to the tumor’s and/or patient’s features. The European Association of Dermato-Oncology (EADO) has recently proposed a new operational classification for daily practice, which comprises easy-to-treat and difficult-to-treat BCCs [6]. Easy-to-treat BCCs encompass all common BCCs that are amenable to surgery and radiotherapy. Difficult-to-treat BCCs include laBCC, mBCC, and common BCCs that pose specific management difficulties and for which surgery and radiotherapy would be inappropriate.

Systemic therapy with hedgehog pathway inhibitors (HHIs) represent the standard of care for difficult-to-treat BCCs [7,8]. Anti-programmed cell death protein 1 (PD-1) antibodies have emerged as a second-line treatment option for aBCC patients who progress or who are intolerant to HHI therapy [9]. A shift in the treatment paradigms toward the neoadjuvant approach is gaining increasing interest in cutaneous oncology, as well as in aBCC management, whereby prior systemic therapy followed by surgery is likely to yield more favorable treatment outcomes, especially when upfront surgical excision could result in permanent functional and/or aesthetic sequelae [10,11].

The aim of the present review is to summarize the systemic treatment strategies currently approved for aBCC and to provide an outlook on the neoadjuvant approach.

## 2. Methods

We performed a non-systematic review of the literature based on PubMed as the search engine and the Clinicaltrials.gov website. No restrictions for publication date, article type, language, and text availability were applied. Keywords employed in the search strategy were as follows: “advanced basal cell carcinoma”, “systemic therapies”, “hedgehog pathway inhibitors”, “immunotherapy”, and “neoadjuvant approach”. The use of neoadjuvant locoregional therapies such as intralesional therapies and radiotherapy was beyond the scope of the present narrative review.

## 3. Results

### 3.1. Approved Systemic Therapies for Advanced BCC

#### 3.1.1. Hedgehog Pathway Inhibitors

HHIs are small molecules the target the smoothened homolog (SMO) protein of the hedgehog signaling pathway [12]. The hedgehog pathway is a key regulator of cell growth and differentiation during embryonic development [13]; however, its aberrant activation during adulthood is the major driver for BCC development and progression [14]. Loss-of-function mutations of the gene encoding for patched homolog 1 (PTCH1) protein account for approximately 90% of the events leading to deregulation of the hedgehog pathway. Less commonly, activating mutations of the *SMO* gene or of the downstream effector glioma-associated oncogene homolog 1 and 2 (GLI) result in uncontrolled activation of the hedgehog signaling cascade [2].

Vismodegib and sonidegib are the two HHIs that have been approved by the Food and Drug Administration (FDA) and the European Medicine Agency (EMA) for the management of aBCC patients who are not candidates for surgery and radiotherapy [15,16].

#### 3.1.2. Vismodegib

Vismodegib was the first HHI to be approved by the FDA and EMA for the management of laBCC and mBCC in 2012 [15]. The pivotal phase II single-arm, two-cohort, multicenter clinical trial ERIVANCE (NCT00833417) investigated vismodegib 150 mg daily in laBCC and mBCC patients; the primary endpoint was the objective response rate (ORR) (complete response (CR) and partial response (PR)) as per independent central review [7]. Data from the long-term extension study (39 months from the completion of accrual) demonstrated an investigator-assessed ORR of 48.5% in the mBCC group (16/33 patients with PR) and 60.3% in the laBCC group (20/63 patients had CR, and 18/63 patients had PR) [17]. Median investigator-assessed progression-free survival (PFS) was 9.3 months (95% CI, 7.4–16.6) for mBCC patients and 12.9 months (95% CI, 10.2–28.0) for laBCC patients.

The open-label, single-arm, multicenter trial STEVIE (NCT01367665) investigated the safety profile of vismodegib in laBCC and mBCC patients [18]. Patients received vismodegib 150 mg daily until disease progression or unbearable toxicity. The primary endpoint was the evaluation of the incidence of adverse events (AEs) until disease progression or occurrence of unacceptable toxicity. Investigators enrolled 1215 patients, of whom 1119 were diagnosed with laBCC and 96 with mBCC. The majority of the study population (*n* = 1192, 98%) experienced at least one AE, the most common being muscle spasms (66%), alopecia (62%), and dysgeusia (55%). At the time of clinical cutoff, vismodegib treatment had been discontinued by 88% of the study cohort, and occurrence of AEs was the most common cause of treatment discontinuation (*n* = 349) [19]. The randomized, regimen-controlled, double-blind, phase II trial MIKIE (NCT01815840) investigated two intermittent vismodegib regimens in patients with multiple BCCs, including basal cell nevus syndrome patients. The primary endpoint was percentage reduction from baseline in the number of clinically evident BCCs at week 73. Investigators reported a significant and similar clinical benefit in both vismodegib intermittent dosing regimens. The safety profile was consistent with previous clinical trials [20].

Several real-life studies confirmed the effectiveness and safety profile of vismodegib, demonstrating an ORR, incidence, and severity of AEs that were comparable to the pivotal trials. The non-interventional study NIELS explored vismodegib effectiveness and safety across 26 German centers in a real-life setting [21]: any grade AE was reported in 95.5% of the enrolled population (63 of 66 laBCC patients), with the most common AEs being muscle spasms (59.1%, 39/66 patients) and alopecia (45.5%, 30/66 patients). The ORR was 74.2% (25/66 CR and 24/66 PR), and the median duration of response was 15.9 months (95% CI: 9.2; 25.7). Accordingly, a multicenter retrospective cohort study from the Netherlands assessed the effectiveness and safety profile of vismodegib in 80 patients (48 with laBCC, 11 with mBCC, and 19 with Gorlin–Goltz syndrome) [22]. The primary endpoint was the median PFS, which was 10.2 months (95% CI, 5.6–22.6), 11.7 months (95% CI, 5.2–17.5), and 19.1 months (95% CI, 7.4–20.2) in laBCC, mBCC, and Gorlin–Goltz syndrome patients, respectively. All patients experienced at least one AE, which were most commonly grade 1–2 in severity (77% of a total of 409 AEs reported in the study cohort). Similar results were reported in a real-world multicenter cohort study on vismodegib effectiveness and safety in 108 aBCC patients (95 with laBCC and 13 with mBCC) from Poland [23]: investigators reported an ORR of 67.6% (19 and 54 patients achieving CR and PR, respectively) and a median PFS of 30.5 months (95%CI 24.8–36.3). The near totality of the patient population experienced at least one AE (83.3%, *n* = 90 patients): muscle spasms was the most common toxicity (*n* = 66 patients, 61.6%), followed by alopecia (*n =* 52 patients, 48.1%).

#### 3.1.3. Sonidegib

Sonidegib was approved by the FDA and EMA for laBCC treatment in 2015 [16]; in Switzerland and Australia, it is also approved for mBCC.

The pivotal phase II multicenter, randomized, double-blind trial BOLT (NCT01327053) investigated the efficacy, safety, and tolerability profile of sonidegib 200 mg and 800 mg in laBCC and mBCC patients not eligible for curative surgery and radiotherapy [8]. The primary efficacy endpoint was the ORR by central review; safety endpoints included AE monitoring and reporting. The study enrolled 230 patients, of whom 79 and 151 were in the 200 mg and 800 mg cohorts, respectively. At the final 42-month analysis, the ORR was 56% for laBCC and 8% for mBCC in the 200 mg group; in the 800 mg group, the ORR was 46.1% and 17% for laBCC and mBCC, respectively [24]. Differently from the ERIVANCE trial, the BOLT trial adopted the modified Response Evaluation Criteria in Solid Tumors (RECIST) version (v) 1.1. (mRECIST), which are more stringent especially with regard to CR assessment [12]. The most common AE was muscle spasms, which occurred in 43/79 (54%) and 104/151 (69.3%) patients in the 200 mg and 800 mg cohorts, respectively. The most common grade 3–4 AE was elevation in creatine kinase (CK), which was reported in 5/79 patients (6%) in the 200 mg group and in 20/151 patients (13.3%) in the 800 mg group [24].

A few real-life studies investigated the effectiveness and safety profile of sonidegib, demonstrating comparable results to the BOLT trial. An observational, retrospective, single-center cohort study from France explored the effectiveness and safety profile of sonidegib in a real-world setting [25]. Investigators enrolled 21 laBCC patients, of whom 6 (29%) patients achieved a CR and 11 (52%) reached a PR, yielding an ORR of 81% (95% CI 59–95%). The entire cohort experienced at least one AE; muscle spasms were the most common AE (*n* = 14; 67%), followed by alopecia (*n* = 12; 57%) and dysgeusia *n* = 8; 38%). A real-life, ambispective, multicenter cohort study investigated the effectiveness and safety profile of sonidegib across 12 tertiary referral centers in Italy [26]. The study enrolled 178 aBCC patients, of whom 85 (*n* = 84 laBCC; *n* = 1 mBCC) had been treated with sonidegib. The primary effectiveness endpoint was ORR, which was 82.1% (95% CI: 88.9–72.6) for laBCC patients (36/84 patients achieving CR, and 34/84 patients achieving PR). Collectively, 168 AEs have been reported in the 85 patients on sonidegib treatment: the majority of the AEs were grade 1–2 in severity (*n* = 139, 82.7%), and muscle spasms (*n* = 51, 30.3%) and dysgeusia (*n* = 45, 26.7%) were the two most common AEs.

#### 3.1.4. Immune-Checkpoint Inhibitors

BCC is a cutaneous malignancy that displays a high tumor mutational burden (>20 mutations/megabase); therefore, it is likely to be responsive to immune-checkpoint inhibitors [27].

Cemiplimab is a humanized monoclonal antibody directed against the PD-1 immune checkpoint, and it is approved as a second-line treatment option for aBCC patients who progress or who are intolerant to HHI therapy [9]. Cemiplimab 350 mg is currently the only immune-checkpoint inhibitor that has been granted FDA and EMA approval in 2021.

A multicenter, single-arm, open-label phase II trial (NCT03132636) investigated cemiplimab 350 mg administered intravenously every 3 weeks in laBCC and mBCC patients who experienced disease progression or were intolerant to prior HHI therapy. The primary endpoint was ORR by independent central review [9]. Data from the extended follow-up of the laBCC cohort (84 patients) revealed an ORR of 32.1%, with 21 PR and 6 CR. Any grade AEs occurred in 83 patients, and the most common AE was fatigue (31%), followed by diarrhea (23.8%) and pruritus (21.4%) [28]. The long-term analysis of the mBCC cohort (54 patients) reported an ORR of 22%, with 10 PR and 2 CR; the most common AEs were fatigue (43%) and diarrhea (37%) [29].

### 3.2. Neoadjuvant Therapies Under Investigation for Advanced BCC

Locoregional treatments for aBCC often have a profound functional and/or aesthetic impact on patients, especially for tumors located in critical areas of the head and neck region as nose and periorificial areas [30]. The neoadjuvant approach is currently shifting the treatment paradigms in cutaneous oncology, as well as in aBCC management. Neoadjuvant therapy is expected to reduce the tumor bulk prior to surgery, with the aim of minimizing the impact of the surgical resection and post-surgical morbidity, as well as improving patients’ quality of life [31]. We provide an overview of the clinical trials investigating HHIs and immune-checkpoint inhibitors in the neoadjuvant setting for aBCC in Table 1.

#### 3.2.1. Hedgehog Pathway Inhibitors

The multicenter, open-label phase II VISMONEO study (NCT02667574) investigated neoadjuvant vismodegib treatment for aBCC of the face deemed inoperable or operable with major aesthetic and/or functional sequelae (Table 1) [32]. Patients received vismodegib 150 mg daily for 4 to 10 months until surgical excision, which was performed at the time of best observed response. Investigators evaluated the complexity of the surgical procedure according to a six-stage surgical risk classification system. The primary endpoint was the proportion of aBCC patients achieving a downstaging in the surgical procedure after neoadjuvant vismodegib compared to baseline. After a median vismodegib treatment duration of 6 months, 44 of 55 patients (80%) reached a better surgical stage compared to baseline, and 27 patients (49%) obtained a clinical CR [32].

The non-randomized, open-label pilot study SONIB (NCT03534947) is currently investigating the efficacy and safety profile of 12 weeks of neoadjuvant sonidegib 200 mg daily followed by surgery or topical imiquimod for the management of BCCs arising on cosmetically challenging locations (Table 1) [33]. The primary endpoint is the tumor response assessed by optical coherence tomography (OCT) at week 12, after the 12-week cycle of neoadjuvant sonidegib. For patients experiencing CR or PR with a superficial remnant lesion, topical treatment with imiquimod will follow; patients with stable disease (SD) or with PR with an invasive remnant lesion will undergo surgical excision.

#### 3.2.2. Immune-Checkpoint Inhibitors

An ongoing single-center, single-arm, open-label phase II study (NCT05929664) is assessing the role of neoadjuvant cemiplimab in laBCC of the head and neck prior to surgery (Table 1) [34]. Patients will receive intravenous cemiplimab 350 mg every 3 weeks for up to six cycles followed by surgical excision, unless experiencing disease progression or unbearable toxicity. The primary endpoint is ORR and disease control rate (DCR) (CR, PR, and SD) by clinical and radiographic assessment according to RECIST v1.1. Secondary endpoints include the pathologic response evaluation; the surgical/clinical benefit rate, defined as the percentage of patients with a tumor response allowing functional organ-preservation surgery, and quality-of-life outcome measures.

Besides cemiplimab, other immune-checkpoint inhibitors are currently being explored in the neoadjuvant setting for aBCC management.

Pembrolizumab is a humanized monoclonal antibody targeting the PD-1 immune checkpoint [35]. A single-arm, open-label phase Ib study (NCT04323202) is evaluating neoadjuvant-adjuvant pembrolizumab in resectable aBCC of the head and neck region (Table 1) [36]. Patients will receive intravenous pembrolizumab 200 mg every 3 weeks for 4 cycles, followed by standard surgical resection and continuation of pembrolizumab in the adjuvant setting for a total of 17 cycles. The primary endpoint is pathologic response defined as change in tumor volume between baseline and surgery as per RECIST v1.1.

Nivolumab is a humanized monoclonal antibody targeting the PD-1 immune checkpoint [37]; it is used in monotherapy or in combination with relatlimab, a humanized monoclonal antibody that functions as an immune-checkpoint inhibitor by binding to the lymphocyte activation gene-3 (LAG3) receptor [38]. A randomized, open-label, phase II clinical trial is currently investigating neoadjuvant nivolumab monotherapy or nivolumab and relatlimab combination in surgically resectable high-risk BCC patients (Table 1) [39]. Patients will be randomized 2:1 to receive intravenous nivolumab 480 mg and intravenous relatlimab 160 mg combination every 4 weeks for up to four cycles or intravenous nivolumab 480 mg monotherapy every 4 weeks for up to four cycles. Both treatment schedules will be followed by surgical resection. Should patients experience disease progression, there is the option to undergo surgical excision after two cycles of neoadjuvant immunotherapy. The primary endpoint is the pathologic response rate, which is defined as the proportion of patients achieving pathological CR (pCR) plus major pathological response (MPR) on the histopathological specimen after tumor surgical resection.

## 4. Discussion

The treatment landscape of BCC has undergone major advances over the last decade, especially with regard to the management of aBCC patients who are not candidates for surgery and radiotherapy [2]. The two HHIs, vismodegib and sonidegib, remain the first-line treatment option for difficult-to-treat BCC, including stage III (laBCC) and stage IV (mBCC), according to the proposed EADO staging system (i.e., stage IIIB including large and destructive tumors in critical areas deemed curable by surgery but with inevitable functional impairment and/or mutilation, or stage IIIC including giant and/or deeply invasive tumors involving extracutaneous tissues for which cure cannot be expected by surgery whatever its extent) [2]. Several real-life studies on sonidegib and vismodegib confirmed the results from pivotal trials [21,22,23,25,26]. The extent of the ORR and the incidence and severity of AEs are in line or sometimes even more favorable in real-life cohorts than in pivotal studies. Also, more complex, elderly, and fragile patient populations were enrolled in real-life studies compared to clinical trials, further underscoring the effectiveness and safety profile of HHIs in a heterogeneous and real-life scenario. The occurrence of class-specific AEs, such as muscle spasms, alopecia, and dysgeusia, as well as the development of resistance to therapy and tumor relapse, represent the main challenges for the long-term management of aBCC patients on sonidegib and vismodegib [40].

Anti-PD-1 immunotherapy with cemiplimab is now the second-line treatment option for aBCC patients who progress or who are intolerant to HHIs [2]. A few case reports explored the effectiveness and safety profile of cemiplimab in complex aBCC patients, confirming the role of immunotherapy in progressing and metastatic aBCCs [41,42,43].

Neoadjuvant therapy followed by surgical excision is expected to yield further progress in the management of difficult-to-treat BCC patients [31]. Besides the published data from the VISMONEO study, a few case series explored the use of neoadjuvant sonidegib or vismodegib followed by surgery in challenging aBCC cases, where upfront surgical excision would have resulted in disfiguring and/or permanent functional sequelae [44,45,46,47]. Radical oncological surgery and preservation of functional and aesthetic outcomes were achieved after a course of neoadjuvant HHI. Concerning immunotherapy, it is assumed to be increasingly effective in the neoadjuvant setting, as T-cells are exposed to a larger and broader tumor antigen repertoire leading to greater T-cell expansion [48,49]. Furthermore, the neoadjuvant regimen would allow for pathologic response evaluation, defined as the percentage of residual viable tumor cells in the surgically resected specimen after neoadjuvant therapy. Evidence from melanoma clinical trials suggests that pathologic response can function as a surrogate of relapse-free survival and overall survival, therefore allowing for prognostication and personalized management, with potential de-escalation of further local and systemic treatments [10].

Several clinical questions are yet to be clarified, such as the optimal duration of the neoadjuvant regimen, the timing of subsequent surgery, and the optimal surgical technique (i.e., microscopically controlled surgery or standard surgical excision). Based on the mechanism of action and the timing of response, a longer treatment course prior to surgery is expected with HHIs such as in the VISMONEO study [32], where patients underwent from 4 to 10 months of vismodegib therapy prior to surgery, and in the SONIB trial [33], with 12 weeks of neoadjuvant sonidegib followed by surgical excision. Shorter treatment duration is expected with immune-checkpoint inhibitors, with two to four cycles of immunotherapy prior to surgery. The extent of surgical excision is another critical point that needs to be elucidated. Neoadjuvant treatment aims to reduce the surgical complexity and to increase tissue preservation; however, tumor-margin mapping may become increasingly difficult after a course of neoadjuvant therapy with persistence of scattered tumoral areas. Emerging imaging techniques such line-field confocal optical coherence tomography (LC-OCT) and high-frequency cutaneous ultrasonography can guide the tumor response assessment during therapy and aid in the pre-surgical evaluation of the tumor margins [50,51,52,53,54,55,56,57].

## 5. Conclusions

Advanced BCC treatment is challenging, especially for tumors located on critical aesthetic and/or functional areas. Systemic therapy with first-line HHIs (vismodegib and sonidegib) and second-line anti-PD-1 immunotherapy (cemiplimab) remain the mainstay of treatment. The neoadjuvant approach based on prior HHIs or immune-checkpoint inhibitors followed by surgery is currently under investigation, and it is expected to revolutionize the management of aBCC patients, leading to downstaging of the surgical procedure, improving post-surgical morbidity and patients’ satisfaction.

## Figures and Tables

**Table 1:** Overview of clinical trials exploring HHIs and immune-checkpoint inhibitors in the neoadjuvant setting for BCC.

NCT	Title of the Study	Phase	Intervention	Status
***Hedgehog pathway inhibitors***				
NCT02667574	Study Evaluating the Interest of Vismodegib as Neo-adjuvant Treatment of Basal Cell Carcinoma (BCC) (VISMONEO)	Phase II	Vismodegib Surgery	Completed
NCT03534947	A Study to Evaluate Neoadjuvant Sonidegib Followed by Surgery or Imiquimod in the Management of Basal Cell Carcinoma (SONIB)	Phase II	Sonidegib Imiquimod Surgery	Recruiting
***Immune-checkpoint inhibitors***				
NCT05929664	Cemiplimab for the Treatment of Locally Advanced Head and Neck Basal Cell Carcinoma Before Surgery	Phase II	Cemiplimab Surgery	Recruiting
NCT04323202	Neoadjuvant-Adjuvant Pembrolizumab in Resectable Advanced Basal Cell Carcinoma of Head&Neck	Phase I	Pembrolizumab Surgery	Active, not recruiting
NCT06624475	Neoadjuvant Opdualag Versus Nivolumab for Resectable High-Risk Basal Cell Carcinoma	Phase II	Nivolumab Relatlimab Surgery	Not yet recruiting

## Data Availability

No new data were created or analyzed in this study. Data sharing is not applicable to this article.
